# Influence of a husband’s healthcare decision making role on a woman’s intention to use contraceptives among Mozambican women

**DOI:** 10.1186/s12978-015-0010-2

**Published:** 2015-04-23

**Authors:** Ramos Mboane, Madhav P Bhatta

**Affiliations:** Department of Biostatistics, Environmental Health Sciences, and Epidemiology, College of Public Health, Kent State University, 750 Hilltop Drive, 319 Lowry Hall, P. O. Box 5190, Kent, OH 44242 USA

**Keywords:** Contraceptive use, Husband or partner’s influence, Healthcare decision making power, Intention to use, Mozambique

## Abstract

**Background:**

Previous studies in developing countries suggest that a husband plays an influential role in a woman’s contraceptive use. The influence of a husband/partner’s healthcare decision making power on a woman’s intention to use contraceptives in Mozambique has not been studied. The present study examined this relationship using data from the 2011 Mozambique Demographic and Health Survey (DHS), which included a nationally representative sample of 7,022 women aged 15-49 years.

**Methods:**

The primary outcome of interest in the study was *a woman’s intention to use contraceptives*. The primary exposure of interest was the person making decisions about a woman’s healthcare, dichotomized as *the husband/partner alone vs. the woman herself or jointly with her husband/partner*. Several potential socio-demographic confounders were adjusted for in overall and stratified multivariable logistic regression models. Adjusted odds ratio (AOR) and the associated 95% confidence interval (CI) are reported.

**Results:**

The mean age of the sample was 30.4 (95% CI: 30.1 - 30.7) years. Overall, a woman who reported her husband/partner usually made the decision about her healthcare was 19% less likely to report an intention to use contraceptives than a woman who reported that she herself or jointly with her husband/partner made the decision (AOR = 0.81, 95% CI 0.71- 0.92). In stratified analyses, the association remained statistically significant among rural women (AOR = 0.75, 95% CI: 0.65 - 0.87); among women with knowledge of modern contraceptive methods (AOR = 0.83, 95% CI: 0.73 - 0.95); and among women with three or more (AOR = 0.81, 95% CI: 0.68 - 0.97) and two or fewer (AOR = 0.79, 95% CI: 0.65 - 0.96) living children.

**Conclusions:**

A husband/partner’s healthcare decision making power in the relationship had a significant negative effect on a Mozambican woman’s intention to use contraceptives. These findings have implications for addressing the role of men in the design and implementation of successful family planning programs to improve the contraceptive uptake rate among women in Mozambique.

## Background

Family planning is regarded as one of the ten greatest public health achievements of the 20^th^ century [1]. Despite the far reaching impact of family planning on the health and well-being of children, women, and families [2-5], modern contraceptive use in the developing world, especially in Africa, remains low [6]. In Mozambique in 2003, the prevalence of any method of modern contraceptive use among women of reproductive age who were married or were in union was 11.8% [6]. Almost a decade later, the 2011 contraceptive use rate in this group of women remained virtually unchanged at 11.5% [7]. Understanding the barriers to modern contraceptive use in Mozambique would assist policy makers and planners in developing targeted family planning programs for increasing the contraceptive use rate.

Women in developing countries face multifaceted and challenging barriers to modern contraceptive use. A complex interplay of demographic, social, cultural, and economic factors contribute to a woman’s failure to use modern contraception. The influence of the male partner on a woman’s reproductive health decisions is an important area of reproductive health research that has garnered greater attention in the last decade [8]. Several previous studies in developing countries suggest that the husband exerts a significant influence on a woman’s decision to use contraceptives [8-10]. Even well-educated women who desire to use contraceptives fail to do so because of their husband’s objection to family planning. For example, in a study from Ghana a husband’s attitude toward family planning was found to strongly influence the wife’s attitude toward contraception [9]. Similar findings were reported in Pakistan, where women faced with making a decision about family planning tend to base their decision on their husband’s fertility preferences and attitudes toward family planning [10].

To the best of our knowledge, there are currently no studies that have examined the role of a husband/partner on a woman’s current contraceptive use or her future intentions of contraceptive uptake in Mozambique. This study aims to understand the influence of a husband/partner’s healthcare decision making power on a woman’s intention to use contraceptives among a nationally representative sample of reproductive aged (15-49 years) women in Mozambique. Quantifying this relationship would provide additional useful data for reproductive health program planning in Mozambique.

## Methods

### Study setting

Mozambique, located in south-eastern Africa, is administratively divided into 11 provinces and 128 districts. The 11 provinces are divided into three geographical regions: the North, the Central, and the South. The population of Mozambique in 2013 was approximately 24 million and women of reproductive age comprised about 24% of the total population. In 2010, the total fertility rate was 5.1 children per woman. The majority of the population in Mozambique is rural (69%) and subsistence agriculture is the main economic activity [11,12]. The healthcare system in Mozambique is predominantly supported by the public sector and consists of the primary (652 health posts and 435 health centers), secondary (27 rural and 8 district hospitals), tertiary (5 general and 7 provincial hospitals), and quaternary (3 central hospitals) levels [13]. While all levels of the public healthcare system provide reproductive health services, mostly free of charge, the primary level plays the most significant role in the promotion and delivery of family planning services.

### Study design and sampling

This cross-sectional study utilized the data from the 2011 Mozambique Demographic and Health Survey (2011 MDHS). The data for the study was down loaded, with permission from the Demographic and Health Survey website at: www.dhsprogram.com/data/available-datasets.cfm. The sampling procedures and survey instrument design have been published in detail previously [7]. Briefly, the 2011 MDHS was a stratified two-stage cluster sampling designed to collect nationally and regionally representative data on population and health indicators. In the first stage, 611 primary sampling units (256 in urban and 355 in rural areas) were identified using a Differential Global Positioning System (DGPS). In the second stage, a representative sample of 13,964 households was randomly selected. Homeless people, and households and individuals living in collective shelters such as hotels, hospitals, military units, and student housing were excluded from this sampling process (this represented an exclusion of 3.3% of the total population). In all 13,871 women of reproductive age (ages 15 – 49 years) eligible for an individual interview were identified from the households selected in the sample and 13,718 of them were interviewed (99% response rate). A total of 7,022 women who were in a union (had a husband or a partner) and had complete information on the primary exposure and main outcome of interest were included in the present analysis.

### Survey instrument and data extraction

The DHS data were collected during face-to-face interviews using tablet computers equipped with a CAPI System (Computer-Assisted Personal Interview) using three questionnaires: the Household, the Women’s, and the Men’s Questionnaire. For the purpose of this study, the Individual Recode Women Dataset derived from the Women’s Questionnaire was used. The Women’s Questionnaire collected data on age, education, religion, reproductive history, knowledge and use of contraceptive methods, antenatal care, marriage and recent sexual activity, fertility preferences, husband’s background, woman’s status, and domestic violence. Portuguese was the language used in the questionnaires, and all the survey instruments were pre-tested in urban and rural areas in the Bilene Macia District of Gaza Province in February 2011. Manual and automatic procedures such as verification of questionnaires, revision and codification, and editing and analysis of inconsistencies were used for data quality assurance and control. Data entry was conducted using microcomputers equipped with Census and Survey Processing System (CSPro) software.

### Primary outcome of interest

The primary outcome of interest in this study was *a woman’s future intention to use modern contraceptives* measured using the question*: are you thinking about using any contraceptive method to delay or avoid getting pregnant in the future?* The possible responses to the question included: *plan to use the methods within the next 12 months*; *plan to use the methods in the future with no time specified; unsure about use*; and *does not intend to use the methods*. These responses were categorized as *those who intended to use the methods in the future*, *those who did not intend to use the methods*, and *those who were unsure about use* in the available MDHS 2011 dataset. Those in the *unsure about use* category were excluded from the present analysis. Thus, the outcome was dichotomized as: those who intended to use contraceptives in the future and those who did not intend to use. To avoid loss of statistical power, observations with missing information on covariates other than the primary exposure and the outcome were not excluded but were treated as missing data.

### Primary exposure of interest

The primary exposure of interest in this study was *the person who usually made the decision on the respondent’s healthcare.* This was designed to capture the information on the individual in the family who had the decision making power in regards to the respondent’s healthcare needs and was measured with the question: *who usually makes the decision about your healthcare?* The possible responses included: *respondent alone*, *husband/partner alone*, *respondent and husband/partner jointly*, *someone else*, and *other*. The latter two groups were excluded in this analysis as they were not related to the research question of interest. The response levels for the primary exposure were dichotomized as: *the respondent alone or jointly with husband/partner* and *the husband/partner alone.* The rationale for combining *respondent alone* and *joint* decision making responses were two-fold: i) there were only a small proportion of the women reporting making the healthcare decision on their own, and ii) the question of interest was whether a husband as a sole healthcare decision maker had an influence on a woman’s contraceptive use intention compared to a decision making process in which a woman was involved.

### Potential confounders

The following potential confounding variables were included in the study: respondent’s age, educational level, employment status, religious beliefs, knowledge about modern contraceptive methods, region of residence, type of place of residence (rural vs. urban); cohabitation status with the husband/partner; number of living children; and husband/partner’s education and desire for children.

### Data analysis

DHS surveys apply the household weights and the individual sampling weights to account for differences in the probability of selection and interview between observations in the study. The use of sampling weights in the analysis is appropriate when calculating representative levels of statistics such as proportions, means, and medians. However, to avoid overestimation of the measure, use of samplings weights is not recommended for estimating relationships such as regression and correlation coefficients [14]. Therefore, the sampling weights [7] were applied to the calculations of proportions and means in the study but not to the logistic regression models. Overall and stratified univariable and multivariable logistic models examined the association between the primary exposure and outcome and the associated odds ratio and 95% confidence intervals (OR; 95% CI) were computed. Data analysis was performed using SAS 9.3 (SAS Institute Inc, Cary, NC, USA) applying SAS Survey procedures (PROC SURVEYFREQ, PROC SURVEYLOGISTIC) to obtain correct estimates and to account for the complex sampling design, when appropriate.

### Ethics statement

This study was conducted using secondary data analysis from the 2011 MDHS dataset. The data collection methods for the 2011 MDHS, including the consent process, have been previously described [7]. Written informed consent for the present analysis was not necessary because secondary data analysis did not involve interaction with the participants. This study was approved by the Kent State University Institutional Review Board as a Level I Exemption Category 4 (Existing Data, Documents, and Specimens) research protocol (#13-578).

## Results

### Sample characteristics

The mean and median ages of the study sample were 30.4 (95% CI: 30.1 – 30.7) and 29.0 (95% CI: 28.6 – 29.5) years. The mean and median number of living children a woman had were 3.0 (95% CI: 2.9 - 3.0) and 2.2 (95% CI: 2.1 - 2.24; range: 0 – 12). Table 1 presents the prevalence of various socio-demographic characteristics and contraceptive use in the study sample. Overall, 38.5% (95% CI: 37.2 – 39.9) of the women reported having no formal education and 41.5% (95% CI: 40.2 – 42.8) reported currently working. Seventy-four (95% CI: 73.4 – 74.3) percent of the women lived in rural areas. Eighty-six percent (95% CI: 84.7 – 86.5) of the women reported that their husband/partner was living with them. Overall, 39.3% (95%: 37.7 – 40.0) of the women reported both she and her husband wanted the same number of children, while 55.2% (95% CI: 53.6 – 56.7) reported that their husband wanted more children than they did. Ninety-six percent (95% CI: 95.5 – 96.3) of the women reported having knowledge of a modern contraceptive method.

**Table 1:** **Sample characteristics of reproductive aged women (15 - 49 years) in the 2011 DHS** ^§^ **-Mozambique**

**Characteristic**	**Frequency**	**Weighted proportion estimate (95% Confidence Interval)**			
		**Overall**	**Person making decisions about the respondent’s health**		
			**Respondent alone or jointly** ^**§§**^	**Husband/ partner alone**	***p - value*** ^*¶*^
		**(N = 7,022)**	**(n = 5,028)**	**(n = 1,994)**	
***Age, years***					0.098
15 – 24	2,182	31.29	30.12	33.68	
		(29.99 - 32.59)	(28.58 - 31.66)	(31.26 - 36.09)	
25 – 34	2,515	35.19	35.85	33.83	
		(33.85 - 36.53)	(34.25 - 37.44)	(31.3 - 36.30)	
35 – 44	1,704	24.51	24.69	24.13	
		(23.29 - 25.72)	(23.25 - 26.12)	(21.87 - 26.40)	
45 – 49	621	9.02	9.34	8.36	
		(8.20 - 9.84)	(8.36 - 10.33)	(6.88 - 9.83)	
***Education***					<0.001
No education	2,524	38.54	36.27	43.18	
		(37.19 - 39.89)	(34.69 - 37.85)	(40.64 - 45.72)	
Primary school	3,686	52.23	52.99	50.65	
		50.83 - 53.62)	(51.35 - 54.65)	(48.06 - 53.23)	
Secondary school or higher	812	9.23	10.73	6.17	
		(8.54 - 9.93)	(9.87 - 11.59)	(5.01 - 7.34)	
***Currently working***					0.003
No	4,285	58.50	56.99	61.57	
		(57.21 - 59.79)	(55.49 - 58.51)	(59.22 - 63.91)	
Yes	2,737	41.50	43.00	38.43	
		(40.21 - 42.79)	(41.49 - 44.51)	(36.09 - 40.78)	
***Religion***					<0.001
Catholic	1,686	28.40	27.54	28.77	
		(27.10 - 29.70)	(26.04 - 29.03)	(26.35 - 31.18)	
Islamic	1,159	19.22	17.45	21.89	
		(18.24 - 20.20)	(16.33 - 18.57)	(20.05 - 23.73)	
Other^*^	3,258	41.85	43.15	37.13	
		(40.64 - 43.07)	(41.71 - 44.60)	(34.96 - 39.30)	
None	776	10.53	10.11	10.89	
		(9.75 - 11.32)	(9.20 - 11.01)	(9.43 - 12.33)	
***Region*** ^**^					<0.001
North	1,723	28.25	25.76	33.34	
		(27.69 - 28.82)	(25.11 - 26.41)	(32.27 - 34.41)	
Central	3,180	53.00	51.01	57.07	
		(52.43 - 53.57)	(50.34 - 51.68)	(56.01 - 58.13)	
South	2,119	18.75	23.23	9.58	
		(18.43 - 19.07)	(22.80 - 23.66)	(9.20 - 9.97)	

^§^DHS: Demographic and Health Survey; ^§§^Jointly = respondent with the husband/partner; ^*¶*^Chi-square p-value testing the differences between women reporting their husband/partner made the healthcare decision along vs. they alone or jointly made the decision; ^*^Other religions include Zion, Evangelical, Protestant, and Anglican; ^**^North region includes the provinces of Niassa, Nampula, and Cabo Delgado; Central region includes the provinces of Zambezia, Tete, Manica, and Sofala; and South region includes the provinces of Inhambane, Gaza, Maputo, and Maputo city.

### Healthcare decision making

Overall, 71.6% of the women in the study reported that they or their partner and they jointly made the decision about their healthcare while 28.4% reported that the husband/partner made the healthcare decision alone. The women who reported that their husband made the healthcare decision were slightly younger than those making the decision jointly (29.62 vs. 30.64 years; p < 0.001.). In addition, a woman’s education level (p < 0.001), employment status (p = 0.003), religion (p < 0.001), region of residence (p < 0.001), urban *vs.* rural residency (p < 0.001), knowledge of modern methods of contraception (p = 0.003), living arrangement with the husband (p < 0.001), husband’s education (p < 0.001), and husband’s desire for children (p < 0.001) were all significantly associated with who made the healthcare decision for the respondent (Table 1).

### Intention-to-use contraceptives

Overall, 44.7% (95% CI: 43.4 – 46.1) of the women reported that they intended to use contraceptives in the future to delay or prevent pregnancy. Among the women who reported that they and their husband jointly make the decision about their health, 46.1% (44.5 - 47.8) reported an intention to use contraceptives in the future, while among women who reported their husbands made the healthcare decision 41.9% (95% CI: 39.4 - 44.4) reported the intention to use contraceptives (*p* = 0.007). In the univariable analysis, intention to use contraceptives was significantly associated with the husband making the healthcare decision, respondent’s age, education level, employment status, religion, knowledge of modern contraceptives, and husband’s educational level and desire for children (Table 2). In the multivariable analysis, a husband making the healthcare decision and a woman’s intention to use contraceptives remained inversely associated (OR = 0.82; 95% CI: 0.72 – 0.93) (Table 3).

**Table 2:** ***Univariable analysis of factors associated with a woman’s intention to use contraceptives*** ^+^ ***, 2011 DHS*** ^§^ ***-Mozambique***

**Factor**	**Odds ratio (95% Confidence Interval)**
*Person making decisions about the respondent’s health*	
Respondent alone or jointly^§§^	1.00
Husband Alone	0.81 (0.73 - 0.89)
*Age, years*	
15 - 24	1.00
25 - 34	0.96 (0.86 - 1.08)
35 - 44	0.45 (0.40 - 0.52)
45 - 49	0.13 (0.11 - 0.17)
*Education*	
Secondary school or higher	1.00
Primary school	0.66 (0.56 - 0.77)
No education	0.45 (0.38 - 0.52)
*Respondent currently working*	
Yes	1.00
No	1.24 (1.13 - 1.37)
*Religion* ^*^	
None	1.00
Catholic	1.00 (0.85 - 1.19)
Islamic	1.04 (0.87 - 1.25)
Other	1.26 (1.07 - 1.47)
*Type of place of residence*	
Urban	1.00
Rural	0.85 (0.77 - 0.95)
*Number of living children*	
Two or fewer	1.00
Three or more	1.01 (0.92 - 1.11)
*Knowledge of modern contraception methods*	
No	1.00
Yes	2.05 (1.58 - 2.66)
*Cohabitation status*	
Husband/partner living elsewhere	1.00
Living with the respondent	0.71 (0.63 - 0.81)
*Husband/partner’s education*	
Secondary school or higher	1.00
Primary school	0.68 (0.60 - 0.77)
No education	0.48 (0.41 - 0.56)
*Desire for number of children*	
Both want the same	1.00
Husband/partner wants more	0.73 (0.65 - 0.82)
Husband/partner wants fewer	0.79 (0.63 - 1.00)

^+^Intention to use contraceptives *vs.* non-intention to use contraceptives in the future; ^§^DHS: Demographic and Health Survey; ^§§^Jointly = respondent with the husband/partner; ^*^Other religions include Zion, Evangelical, Protestant, and Anglican.

**Table 3:** **Association of a husband/partner’s healthcare decision making role** ^**+**^ **and a woman’s intention to use contraceptives** ^**++**^ **, 2011 DHS** ^ƗƗƗ^ **-Mozambique**

**Logistic regression model**	**Odds ratio (95% Confidence Interval)**			
	**Unadjusted**		**Adjusted**	
*Overall* ^*¶*^ *(n = 7,022)*	0.81	(0.73 - 0.90)	0.82	(0.72 - 0.93)
*Stratified by:*				
Place of residence*				
Urban (n = 2,145)	0.97	(0.79 - 1.18)	1.00	(0.75 - 1.34)
Rural (n = 4,877)	0.76	(0.68 - 0.86)	0.75	(0.65 - 0.87)
Knowledge of modern contraceptive methods**				
No (n = 278)	0.31	(0.17 - 0.58)	0.82	(0.34 - 2.00)
Yes (n = 6,737)	0.84	(0.75 - 0.93)	0.82	(0.72 - 0.94)
*Number of living children* ^*§*^				
Two or less (n = 3,418)	0.77	(0.66 - 0.89)	0.79	(0.65 - 0.95)
Three or more (n = 3,604)	0.84	(0.73 - 0.97)	0.81	(0.68 - 0.97)
*Desire for number of children* ^§§^				
Both want the same (n = 2,237)	0.96	(0.79 - 1.17)	0.97	(0.79 - 1.20)
Husband/partner wants more (n = 2,650)	0.77	(0.66 - 0.91)	0.70	(0.58 - 0.83)
Husband/partner wants fewer (n = 343)	0.72	(0.44 - 1.19)	0.77	(0.44 - 1.36)

^+^Husband/partner making decision solely *vs.* Woman alone or woman and husband/partner jointly.

^++^Intention to use contraceptives *vs.* non-intention to use contraceptives in the future.

^ƗƗƗ^DHS: Demographic and Health Survey.

^*¶*^Multivariable model adjusted for: Respondent’s age, education level, working status, religion, region and type of place of residence, knowledge of any contraceptive method, number of living children, cohabitation status, desire for number of children and husband/partner’s education.

^*^Multivariable model adjusted for all the variables listed above except place of residence.

^**^Multivariable model adjusted for all the variables listed above except knowledge of modern contraceptive methods.

^*§*^Multivariable model adjusted for all the variables listed above except number of living children.

^*§§*^Multivariable model adjusted for all the variables listed above except the desire for children.

In the stratified analysis by the type of place of residence, the inverse relation between the husband making the healthcare decision for the respondent and a woman’s intent to use contraceptives remained only among rural women (OR = 0.75; 95% CI: 0.65 – 0.87) (Table 3). Similarly, the relationship held when assessed only among women who knew of modern contraceptive methods (OR = 0.82; 95% CI: 0.72 – 0.94). The negative relationship between a husband making healthcare decisions for a respondent and a woman’s intention to use contraceptives remained statistically significant regardless of the number of living children. However, in terms of the husband’s desire for children, the relationship only held among women who reported that their husband wanted more children (OR = 0.70; 95% CI: 0.58 – 0.83) than them (Table 3).

## Discussion

In this study conducted in a nationally representative sample of reproductive aged women in Mozambique, we observed that a majority of the women reported that they did not intend to use contraceptives in the future. Furthermore, those women who indicated that their husband made healthcare decisions for them were 18% less likely to report an intention to use contraceptives. The indication by the majority of the women surveyed that they did not intend to use contraceptives in the future poses a tremendous challenge for increasing the current low contraceptive use rates in Mozambique. Increasing the contraceptive uptake in Mozambique is necessary for impacting the country’s high fertility, infant mortality, and maternal mortality rates. The healthcare decision making power of a husband as a significant barrier for a woman’s intent to use contraceptives observed in this study highlights the influence that males exert on the reproductive health decisions of Mozambican women. These findings have implications for future reproductive health program planning in the country.

The results of our study are similar to findings from other developing countries that have assessed the role of males in reproductive health decision making for women. For example, a 2010 study in Cambodia reported that women who agreed with the statement that their husband was the one who makes the final decision about contraception were 50% less likely to be practicing contraception than women who disagreed with that statement [15]. Similarly, in a study in Pakistan, a woman’s inability to discuss family planning issues with her husband negatively affected her intention to use contraception (OR = 0.81) and a woman’s perception that the husband was the sole decision-maker on family planning issues negatively affected a woman’s intention to use contraception (OR = 0.74) [16]. Although the socio-economic, cultural, religious and political contexts of the study settings vary, consistent association of the husband’s healthcare decision making power negatively impacting a woman’s contraceptive use provides important insight for family planning and reproductive health program planners. The role and influence of husbands need to be taken into account when developing family planning services and programs for women to increase contraception in developing countries.

The association between the husband/partner making the healthcare decision for the woman and her intention to use contraceptives was observed among rural women, but not among urban women. This may be due to the fact that family planning services are more likely to be available in urban than in rural areas, and women in urban areas are more likely to be aware of these services and their benefits. Moreover, urban women are more likely to be educated, employed and empowered, allowing them to make independent choices about their health without overly -relying on their partners. While data from Mozambique are limited on these observations, studies from other countries have found a consistent relationship between a woman’s education, employment status, socioeconomic status, and place of residence (urban vs. rural) and their utilization of modern contraception [15-18]. Our study findings highlight the modifying effect of place of residence (rural *vs.* urban), which is a proxy for socioeconomic and cultural factors, and contraceptive accessibility, on the relationship between a woman’s intention to use contraceptives and the husband’s healthcare decision making role in the relationship.

The inverse relationship between a husband’s healthcare decision making and a woman’s intent to use contraceptives remained only among women with knowledge of modern contraceptives when stratifying by the contraceptive knowledge. These findings suggest that besides having knowledge of modern contraceptive methods, another factor that drives the women’s decision for not engaging in contraceptive use in Mozambique appears to involve the male subject (husband/partner). Additionally, the husband’s desire for more children appears to negatively influence a woman’s intention to use contraceptives; among women who reported that the husband wanted more children than them, women whose healthcare decisions were solely made by their husband/partner were 30% less likely to use contraceptives as compared to those who made the decision themselves or jointly with their husband/partner. The fact that the association between a husband’s healthcare decision making and a woman’s intent to use contraception remained regardless of the number of living children suggests that even though the woman has the knowledge of modern contraceptive methods and has the desire to limit the family size, she is less likely to use contraception due to her husband making the decision for her.

This study did not specifically assess how and why the healthcare decision making power by the male in the relationship influences a woman’s intent to use contraception among Mozambican women. However, the existing body of research from other African countries may provide some insight into the possible reasons. A 2014 study from Uganda found that in rural areas men were the primary decision-makers at the household level and acted as obstacles to women’s utilization of family planning services [18]. These men, as the financial managers of the family assets, tend to perceive modern contraceptives as an additional cost for the family because of the contraceptive purchase costs and expenses associated with the treatment of side effects of contraceptives. The researchers of the study posit that these financial barriers need to be realistically taken into account when designing strategies that seek to change men’s attitudes towards the use of modern contraceptives. Future research is needed to explore if these and/or other reasons may explain how a healthcare decision hinders a woman’s intention to use contraception in Mozambique.

For Mozambique to increase contraceptive use among women of reproductive age, the barrier of a male’s influence on a woman’s intention to use contraceptives must be addressed. Various strategies have been designed and implemented to improve male involvement in family planning and contraceptive use decision making in other countries that perhaps could be adapted for application in Mozambique. For example, a program in Kenya successfully used workplace motivators to educate males about contraception, birth spacing and other reproductive health matters [19]. Another initiative implemented in Benin by local organizations used dramas to persuade men to be more supportive of their wives’ desires towards contraception. This approach contributed to a significant decrease in the numbers of children desired by both men and women in Benin [20]. Such innovative approaches need to be explored in Mozambique to engage men in a positive reproductive health decision making process and change them to facilitators for women’s contraceptive use.

Our study has several strengths and limitations. This study included a nationally representative sample thus has high generalizability. Moreover, this study used the most recent DHS data available and thus provides the current situation in Mozambique. Most of the prior studies using the DHS data have grouped Mozambique with other Sub-Saharan African countries. However, our study was conducted using cross-sectional survey data, and therefore does not allow for any causal inferences between the main exposure and the outcome. The self-reported nature of the exposure and outcome may result in misclassification bias. However, any misclassification is likely to be non-differential, thus the observed association is likely an underestimation of the actual. We were also unable to ascertain the potential impact of unmet contraceptive needs. We were unable to adjust for the respondent’s past experience with contraceptives use, which may have a bearing on the intention to use or not use in the future. Finally, the outcome assessed in the study was the intention to use contraceptives in the future, which may not accurately predict the actual use.

## Conclusion

In summary, this study found that among a nationally representative sample of Mozambican women of reproductive age (15 – 49 years old), there was a significant effect from the husband/partner’s healthcare decision making power on women’s intentions to use contraceptives, especially among rural women, regardless of the number of living children. These findings support the call for targeting males for their greater involvement in reproductive health programs and initiatives being implemented in Mozambique. Male involvement in the family planning decision making process is likely to have an impact on Mozambique reaching its target goal of increasing contraception utilization by women of reproductive age. This is vital for the country as family planning is an effective public health tool that guarantees that women “stay healthier, are more productive, and have more opportunities for education, training, and employment, which in turn, benefits entire families, communities and nations” [21].

## References

[1] Centers for Disease Control and Prevention (CDC): Ten great public health achievements -- United States, 1900 – 1999. MMWR Morb Mortal Wkly Rep. 1999;48:241–243. 10220250

[2] Singh S, Darroch JE, Ashford LS, Vlassoff M: Adding it up: the benefits of investing in sexual and reproductive health care. New York: The Alan Guttmacher Institute (AGI) and United Nations Population Fund (UNFPA); 2003.

[3] Centers for Disease Control and Prevention (CDC): Family Planning Methods and Practice: Africa. Second Edition. Atlanta, Georgia: United States Department of Health and Human Services, Centers for Disease Control and Prevention, National Center for Chronic Disease Prevention and Health Promotion, Division of Reproductive Health; 2000.

[4] Peterson HB, Darmstadt GL, Bongaarts J (2013). Meeting the unmet need for family planning: now is the time. Lancet.

[5] Cleland J, Bernstein S, Ezeh A, Faundes A, Glasier A, Innis J (2006). Family planning: the unfinished agenda. Lancet.

[6] United Nations: World contraceptive use 2011 [http://www.un.org/esa/population/publications/contraceptive2011/wallchart_front.pdf]

[7] Ministerio da Saude (MISAU), Instituto Nacional de Estatística (INE) e ICF International (ICFI). Moçambique Inquérito Demográfico e de Saúde 2011. Calverton, Maryland, USA: MISAU, INE e ICFI. [http://dhsprogram.com/pubs/pdf/FR266/FR266.pdf]

[8] Bankole A, Singh S (1999). Couple’s fertility and contraceptive decision – making in developing countries: hearing the man’s voice. Int Fam Plan Perspect.

[9] Akinrinola B (1995). Desired fertility and fertility behavior among the Yoruba of Nigeria: a study of couple preferences and subsequent fertility. Popul Stud (Camb).

[10] Casterline JB, Sathar ZA, Huque UM (2001). Obstacles in contraceptive use in Pakistan: a study in Punjab. Stud Fam Plann.

[11] The World Health Organization: Mozambique: health profile [http://www.who.int/gho/countries/moz.pdf?ua=1]

[12] The World Fact Book [https://www.cia.gov/library/publications/the-world-factbook/]

[13] de Moçambique R (2006). Plano de Acção para Redução da Pobreza Absoluta.

[14] Rutstein SO, Rojas G (2006). Guide to DHS Statistics – Demographic and Health Surveys Methodology.

[15] Samandary G, Speizer IS, O’Connell K (2010). Role of social support and parity on contraceptive use in Cambodia. Int Perspect Sex Reprod Health.

[16] Agha S (2010). Intentions to use contraceptives in Pakistan: Implications for behavior change campaigns. BMC Public Health.

[17] Lakew Y, Reda AA, Tamene H, Benedict S, Deribe K (2013). Geographical variation and factors influencing modern contraceptive use among married women in Ethiopia: evidence from a national population based survey. Reprod Health.

[18] Kabagenyi A, Jennings L, Reid A, Nalwadda G, Ntozi J, Atuyambe L (2014). Barriers to male involvement in contraceptive uptake and reproductive health services: a quality study of men and women’s perceptions in two rural districts in Uganda. Reprod Health.

[19] Center for Communication Programs, Johns Hopkins School of Public Health: Reaching Men Worldwide: Lessons Learned from Family Planning and Communication Projects - 1986-1996. Working Paper Series No.3. Baltimore, Maryland: Johns Hopkins University School of Public Health, Center for Communication Programs; 1997.

[20] Greene ME, Mehta M, Pulerwitz J, Wulf D, Bankole A, Singh S: Involving men in reproductive health: contributions to development [http://www.unmillenniumproject.org/documents/Greene_et_al-final.pdf]

[21] Obaid TA: Invest in the health of women. Invest in sexual and reproductive health [http://www.un.org/en/ecosoc/president/corner/UNFPA.pdf]

